# Experimental and theoretical evidence for unprecedented strong interactions of gold atoms with boron on boron/sulfur-doped carbon surfaces†‡

**DOI:** 10.1039/d3na00956d

**Published:** 2023-12-11

**Authors:** Samya Banerjee, Juliusz A. Wolny, Mohsen Danaie, Nicolas P. E. Barry, Yisong Han, Houari Amari, Richard Beanland, Volker Schünemann, Peter J. Sadler

**Affiliations:** a Department of Chemistry, University of Warwick CV4 7AL Coventry UK P.J.Sadler@warwick.ac.uk; b Department of Chemistry, Indian Institute of Technology (BHU) Varanasi Uttar Pradesh 221005 India samya.chy@itbhu.ac.in; c Department of Physics, University of Kaiserslautern-Landau Erwin-Schrödinger-Straße 46 67663 Kaiserslautern Germany wolny@rhrk.uni-kl.de; d Diamond Light Source Ltd, electron Physical Science Imaging Centre (ePSIC), Harwell Science & Innovation Campus Didcot Oxfordshire OX11 0DE UK mohsen.danaie@diamond.ac.uk; e School of Chemistry and Biosciences, University of Bradford Bradford BD1 7DP UK; f Department of Physics, University of Warwick Gibbet Hill Road Coventry CV4 7Al UK

## Abstract

The 16e square-planar bis-thiolato-Au(iii) complexes [Au^III^(1,2-dicarba-*closo*-dodecarborane-1,2-dithiolato)_2_][NBu_4_] (Au-1) and [Au^III^(4-methyl-1,2-benzenedithiolato)_2_][NBu_4_] (Au-2) have been synthesized and fully characterized. Au-1 and Au-2 were encapsulated in the symmetrical triblock copolymer poloxamer (Pluronic®) P123 containing blocks of poly(ethylene oxide) and poly(propylene oxide), giving micelles AuMs-1 and AuMs-2. High electron flux in scanning transmission electron microscopy (STEM) was used to generate single gold atoms and gold nanocrystals on B/S-doped graphitic surfaces, or S-doped amorphous carbon surfaces from AuMs-1 and AuMs-2, respectively. Electron energy loss spectroscopy (EELS) data suggested strong interactions of gold atoms/nanocrystals with boron in the B/S-doped graphitic matrix. Density-functional theory (DFT) calculations, also supported the experimental findings, pointing towards strong Au–B bonds, depending on the charge on the Au–(B-graphene) fragment and the presence of further defects in the graphene lattice.

## Abstract 2

Single Au atoms and Au nanocrystals on doped graphitic surfaces generated by beam irradiation of micelle-encapsulated Au complexes in an aberration-corrected scanning transmission electron microscope, exhibit strong Au–B interactions.

## Graphical abstract

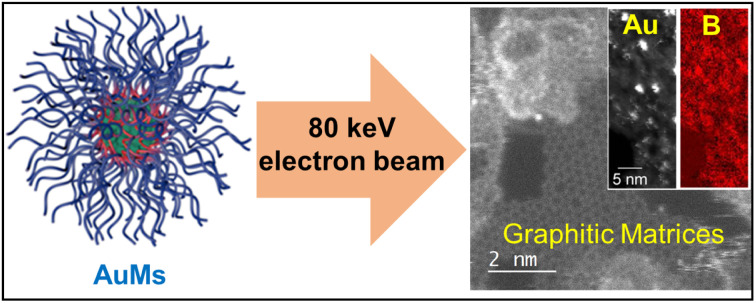

## 1. Introduction

Gold nanocrystals have many potential applications ranging from therapy and diagnosis to catalysis and circuitry.^1–6^ Understanding the process of nanocrystal formation at the atomic level is a topical area of research.^7–10^ The fabrication of nanocrystal matrices is of significant importance for the development of nanodevices.^11,12^ Aberration-corrected electron microscopy offers the possibility of detecting and studying single-atom dynamics, but elucidating the interactions of heavy metal single atoms/nanocrystals with the underlying nanofabricated carbon matrices at the atomic level is a major challenge.^13–15^ The combination of these two challenges in nanocrystal chemistry (matrix fabrication and interaction of heavy metal single atoms/nanocrystals with the fabricated matrices) is addressed in the present work.

Previously, we utilized electron beam irradiation in an aberration-corrected transmission electron microscope for generating single metal atoms and nanocrystals from polymer-encapsulated precious metal complexes.^14–18^ In this work, we use scanning transmission electron microscopy (STEM), and in particular the very high electron flux from beam irradiation, to induce chemical reactions of gold(iii) complexes encapsulated in polymeric micelles spread across the holes of lacey carbon sample support grids. We show that the surface produced can be either a boron–sulfur-doped graphitic surface or an amorphous carbon surface, depending on the nature of the gold-coordinated ligand. Although Au–B σ bonds and 3c-2e B–Au–B bonding have been reported within a pure Au–B alloy,^19^ as well as a gold boride complex with fluxional Au–B bonding,^20^ there appear to be no precedents for gold–boron interactions within boron/sulfur-doped graphitic matrices, as observed here by electron energy loss spectroscopy (EELS) studies. The occurrence of such Au–B interactions is supported by DFT calculations.

## 2. Results and discussion

### 2.1. Preparations, STEM and EELS data

**Scheme 1:**
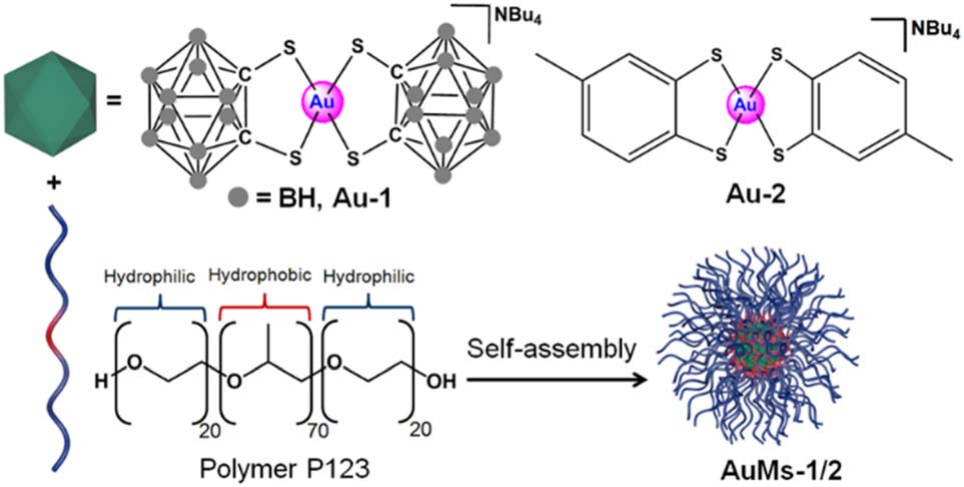
Synthesis of AuMs-1 and AuMs-2 by self-assembly of block copolymer (P123) micelles containing encapsulated 16e Au(iii) complexes [Au(1,2-dicarba-*closo*-dodecarborane-1,2-dithiolato)_2_][NBu_4_] (Au-1) or [Au(4-methyl-1,2-benzenedithiolato)_2_][NBu_4_] (Au-2).

We compared the two 16e square-planar bis-thiolato-Au(iii) complexes Au-1 and Au-2 that differ only in the presence of carboranyl or tolyl groups as substituents on chelated 1,2-dithiolato ligands. Both gold complexes are highly hydrophobic, but become water-dispersed once encapsulated in the symmetrical triblock copolymer poloxamer (Pluronic®) P123 containing blocks of poly(ethylene oxide) and poly(propylene oxide) (Scheme 1).

Encapsulation of [Au(1,2-dicarba-*closo*-dodecarborane-1,2-dithiolato)_2_][NBu_4_] (Au1) led to the isolation of purple-red micelles with a very low-dispersity parameter.^18^ P123 micelles form stable Langmuir films at room temperature and are deformable on surfaces.^21^ Hence, we deposited aqueous AuMs-1 droplets ([AuMs-1] = 1 mg ml^−1^) onto lacey carbon TEM grids to generate an unsupported film over the grid holes for study by aberration-corrected scanning transmission electron microscopy.

**Fig. 1:**
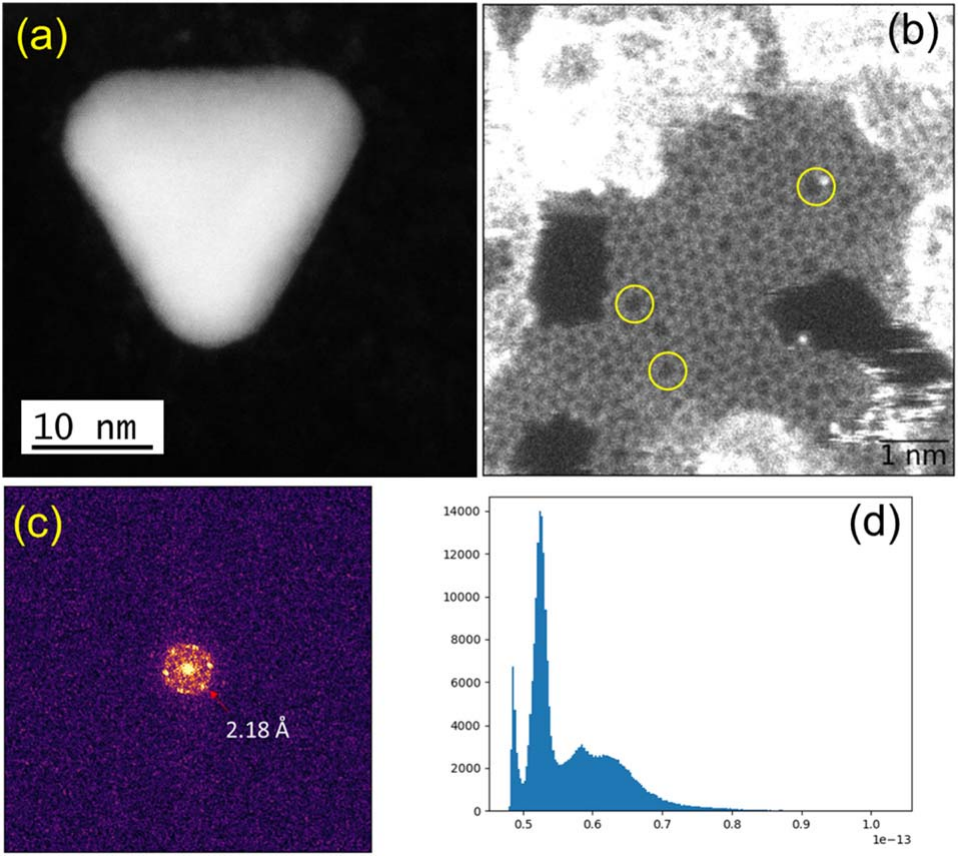
(a) Gold crystal generated from AuMS-1 by electron beam irradiation. (b) Raw HAADF image with FOV of 6.8 nm showing the formation of graphitic matrices upon beam irradiation and topological defects (yellow circles) within the graphitic matrices. (c) FFT of the masked image (hanning window applied to remove edge effects), corresponding to a lattice spacing of 2.18 Å, that of the (100)/(010) planes of graphite. (d) Histogram of the raw image after applying a Gaussian blur (with 2 pixels kernel) showing that the annular dark-field signal is not saturating with the imaging conditions used.

We observed structural changes within the high-energy electron beam irradiation region of this grid on a JEOL ARM 200F microscope using an accelerating voltage of 80 keV. We illuminated an area roughly equal in size to a grid-bar region using a stationary electron beam defined by a 150-μm aperture with total beam current of 9.6 nA for 15 minutes. Such irradiation causes rapid C–H and other bond dissociations.^22^ STEM imaging showed the formation of Au-nanoclusters with sizes varying from single atoms to *ca.* 9 nm diameter (Fig. 1(a) and S1‡) on a graphitic structured surface (Fig. 1(b)) after 5 min of beam irradiation. The measured Au(111) *d*-spacing was 2.35 Å, consistent with that of bulk gold (Fig. S2‡).^23^ The spots in the fast Fourier transform (FFT) for the region in Fig. 1(b) correspond to a lattice spacing of 2.18 Å, the (100)/(010) planes of graphite (Fig. 1(c)).^24^ The histogram of the raw image (Fig. 1(d)) shows that the annular dark-field signal is not saturating with the imaging conditions used. The main framework consists of 6-membered C/B rings, alongside visible defects in the regular array. Fig. 1(b) shows many topological defects (Stone–Wales defects).^25,26^ Such defects are commonly observed in graphene samples.^25,26^ These increases in ring size are likely to be due to the presence of S in the lattice, which is considerably larger in size than C and B, causing expansion and irregular ring shapes scattered through the structure.

**Fig. 2:**
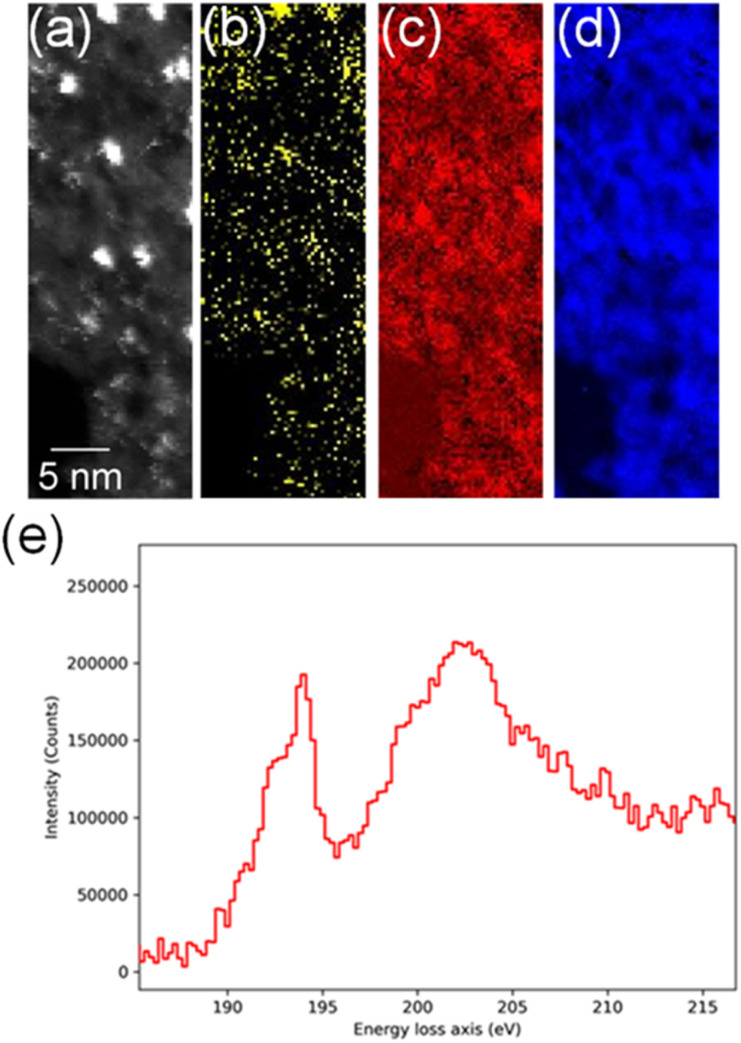
(a) High-angle annular dark-field (HAADF)-STEM image showing Au nanocrystals, (b) EELS S–L_2,3_ map (165.5–189.3 eV), (c) EELS B–K map (186.5–213.3 eV), (d) EELS C–K map (280.5–336.8 eV), (e) EELS data showing the fine spectral features for boron within the sulfur-doped boronic graphitic surfaces.

In order to confirm the simultaneous presence of C, B and S in the matrix, electron energy loss spectroscopy (EELS) was used to characterise the graphitic material. The result clearly indicates that all three elements are present (Fig. 2(a)–(d), and S3‡). The relatively sharp edge at *ca.* 194.0 eV and broader peak centred at *ca.* 203 eV (Fig. 2(e)) on the B K-edge indicate that all the boron atoms are sp^2^ hybridized in a planar bonding configuration within the graphite-like structure.^27^ These features are in good agreement with the B K-edge spectrum of hexagonal boron nitride, for which a 
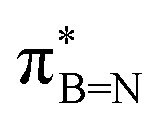
 peak at 192.0 eV and 
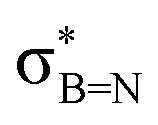
 peak at ∼199 eV are observed.^27^ Interestingly, the B K-edge signal also resembles well that reported for B_2_O_3_.^28^ Involvement of sp^2^ hybridized carbon in the formation of the graphite-like matrices was clearly evident from the C K-edge with a sharp π* feature at *ca.* 286 eV (Fig. S4‡). Principal component analysis (PCA) was carried out to further support the simultaneous presence of C, B, and S in the graphitic matrices. The “scree plot” (Fig. S5‡) obtained from the PCA provides an indication of how many components contribute the most to the EELS data. Here we have chosen 7 components (Fig. S5‡). The first component is the average of the dataset. Component-2 is dominated by the C–K edge. The B–K edge is captured in component-4. Moreover, the density of B is higher in the regions rich in Au nanocrystals, as observed in the corresponding HAADF STEM image.

**Fig. 3:**
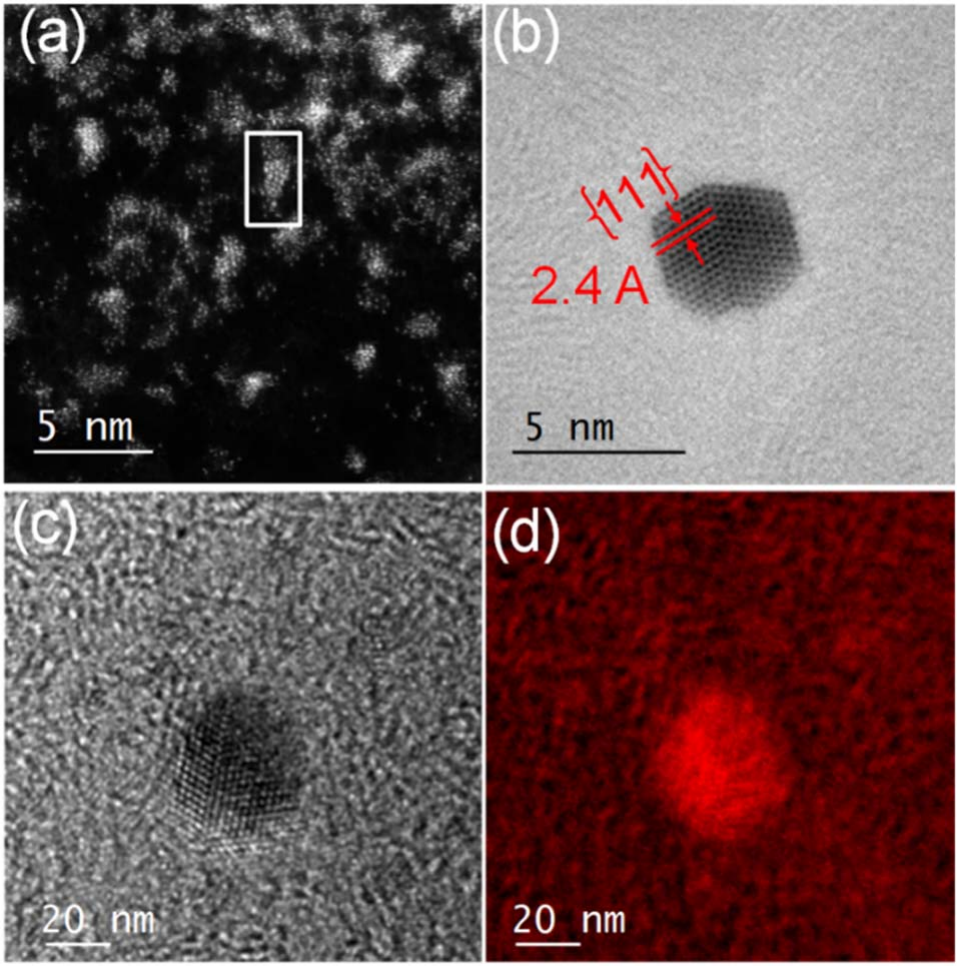
(a) STEM- HAADF image showing Au-clusters with size varying from single atoms to *ca.* 2.7 nm diameter generated from AuMs-2. (b) HRTEM image showing specific surface elemental rearrangements of Au clusters leading to the formation of a well faceted nanocrystal. (c) and (d) EFTEM maps showing that the nanoclusters generated from AuMs-2 are primarily composed of Au.

In contrast, encapsulation of [Au(4-methyl-1,2-benzenedithiolato)_2_][NBu_4_] (Au-2) led to the isolation of green micelles (AuMs-2) with a low-dispersity parameter (*ca.* 0.3) and a hydrodynamic diameter which increased from 19.6 ± 1.8 nm to 195.1 ± 10.4 nm (Fig. S6‡). Gold content, determined by ICP-MS analysis, indicated the presence of *ca.* 112 ± 24 molecules of Au-2 per micelle. On deposition of aqueous AuMs-2 droplets ([AuMs-2] = 1 mg ml^−1^) onto a lacey carbon-coated TEM grid followed by electron beam irradiation (80 keV) of *ca.* 9.6 nA for 15 min on an area roughly equal in size to a grid-bar region, many clusters of gold atoms, ranging in size single atoms to *ca.* 2.7 nm diameter nanocrystals, were observed on the surface (Fig. 3(a)). The average Au–Au distance of 0.26 ± 0.01 nm (122 measurements, Fig. S7‡), is consistent with that of bulk gold.^29^ Complementary high-resolution transmission electron microscopy (HRTEM) studies tracking the same individual Au nanoclusters under 10 min of beam irradiation in a JEOL 2100 TEM microscope operating at 200 keV, showed highly specific surface elemental rearrangements of these clusters (Fig. 3(b)), and the formation of well faceted nanoparticles. The lattice spacing of 2.4 Å corresponding to the {111} planes is in good agreement with the literature.^23^ As clearly shown in movie-1 (see supporting file‡), these clusters are of different sizes ranging from 2 to 4 nm and can be regarded as resulting from coalescence of smaller particles. Furthermore, the movie confirms the presence of twinning (Fig. 3(b), which can be attributed to the coalescence of two or more small crystals).

For elemental identification, energy-filtered TEM (EFTEM) images were acquired (Fig. 3(c) and (d)) by employing the “jump ratio” method of the Au O edge using 30 eV energy windows for pre- and post-edge images, energy slit width of 20 eV, exposure time of 30 s, and total integration time of 90 s. The EFTEM maps clearly show that the nanoclusters are primarily composed of Au.

### 2.2. DFT modelling

#### 2.2.1. Graphene doped with one boron atom

**Fig. 4:**
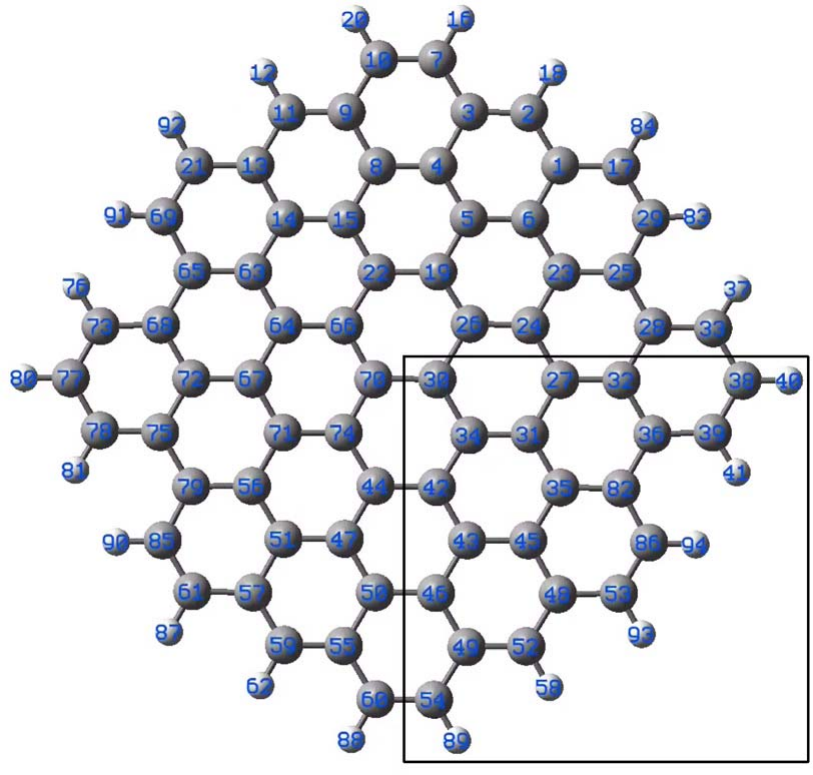
Model graphene surface fragment 1 used for the DFT modelling. The atoms C27, C30–C36, C42–C43, C46, C48-49-49 and C82 were substituted with a B atom for each model.

In order to obtain insights into possible bonding of gold to the B-doped graphene, DFT modelling was performed. Graphene was modelled with the C_72_H_22_ molecule shown in Fig. 4. The molecule displays *C*_2v_ symmetry consisting of four equivalent parts. Modelling was performed for the fragment marked with the black rectangle. Each carbon was replaced with a boron atom, apart from those on the rim bound to hydrogen, which are assumed to be less relevant for the system investigated in the experiment. Thus, 14 different C_71_BH_22_ molecules were used for the modelling as listed in Table 1. The first part of the calculations considered AuC_71_BH_22_ models. We performed optimizations of the geometry of the isolated molecule followed by frequency calculations using the TPSS functional^30^ and QZVP basis set.^31^ The Gaussian 16 (ref. 32) package was used. For each B-substitution four different isomers were considered: (i) 533-electron molecule with a doublet ground state corresponding formally to a negatively charged C_71_BH_22_ fragment bound to Au(0) (ii) triplet and singlet states of 532-electron molecules corresponding formally to the neutral C_71_BH_22_ fragment bound to Au(0) with the doped-graphene fragment and gold coupled ferromagnetically and antiferromagnetically, respectively. Note that the diamagnetic isomer may also represent the complex of negatively-charged C_71_BH_22_ bound to Au(i). (iii) 531-electrons molecule with a doublet ground state, corresponding formally to the neutral AuC_71_BH_22_ fragment bound to Au(0).

**Table 1:** Calculated energies of Au–B bond formation for the modelled AuC_71_BH_22_ species and Au_5_C_71_BH_22_. For comparison, the values obtained for the systems with gold bound to C27 are given. The Au–C distances and C–B–Au angles are given when the Au–B coordination is not axial, *i.e.* the Au–B bond is not perpendicular to the graphene plane

Atom bound to gold	Energy of Au-adduct formation (kJ mol^−1^)				Geometry of coordination (angles in deg.) and bond distances (Å)							
	Molecule, number of electrons, spin state				Geom. B–C	Au–B	Geom. B–C	Au–B	Geom. B–C	Au–B	Geom. B–C	Au–B
	Au(0)L^−^ 533 doublet	Au(0)^−^L 532 triplet	Au(0)L 532 singlet	Au(i)L 531 doublet	Au(0)L^−^ 533 doublet		Au(0)^−^L 532 triplet		Au(0)L 532 singlet		Au(i)L 531 doublet	
B27	−73	−72	−127	−505	Axial	2.282	Axial	2.266	Axial	2.190	Axial	2.185
B30	−71	−65	−120	−499	Axial	2.287	Axial	2.272	Axial	2.188	Axial	2.183
B31	−63	−56	−95	−480	Axial	2.262	Axial	2.273	C34–Au 2.509	2.217	C27–Au 2.398	2.224
B32	−80	−66	−128	−507	Axial	2.295	C27–B–Au 99	2.282	C27–B–Au 100	2.185	C27–B–Au 100	2.183
B34	−74	−61	−115	−486	Axial	2.275	C42–Au 2.459	2.305	C31–Au 2.354	2.242	C31–Au 2.586	2.191
B35	−76	−60	−127	−498	Axial	2.291	Axial	2.266	C82–Au 2.219	2.314	C82–Au 2.258	2.259
B36	−102	−72	−156	−529	C39–Au 2.183	2.559	C82–Au 2.383	2.315	C39–Au 2.145	2.387	C39–Au 2.170	2.309
B42	−79	−57	−115	−481	Axial	2.261	C34–Au 2.470	2.304	C44–Au 2.234	2.253	C44–Au 2.377	2.226
B43	−74	−63	−117	−496	Axial	2.288	C42–Au 2.541	2.293	C46–Au 2.403	2.216	C46–Au 2.380	2.213
B45	−74	−62	−119	−496	Axial	2.288	C35–Au 2.569	2.283	Axial	2.189	Axial	2.184
B46	−78	−61	−120	−491	Axial	2.268	C49–Au 2.497	2.294	C50–Au 2.520	2.205	Axial	2.185
B48	−101	−102	−167	−530	C52–Au 2.223	2.419	C86–Au 2.407	2.229	C86–Au 2.407	2.229	C52–Au 2.164	2.303
B49	−104	−77	−166	−532	C52–Au 2.312	2.342	C54–Au 2.263	2.378	C52–Au 2.140	2.392	C52–Au 2.148	2.34
B82	−90	−68	−118	−492	Axial	2.251	C86–Au 2.279	2.374	C86–Au 2.407	2.229	C86–Au 2.225	2.294
C27	−19	−18	−43	−420	Axial C–B	2.397	Axial C–B	2.378	Axial C–B	2.158	Axial C–B	2.151
B30	Au_5_(0)L^−^ 849 doublet	Au_5_(0)L 848 triplet	Au_5_(0)L 848 singlet	(Au_5_)^+1^(0) L 847 doublet	Au_5_(0)L^−^ 849 doublet		Au_5_(0)L 848 triplet		Au_5_(0)L 848 singlet		(Au_5_)^+1^(0) L 847 doublet	
	−91	−71	−88	−261	Au–C44	Au–B30	Au–C26	Au–B30	Au′–C44	Au–B30	Au′–C44	Au–B30
					2.589	2.335	2.386	2.348	2.530	2.299	2.568	2.284

The results of the calculations showing the obtained geometries and estimated binding energies of Au–B bonding are collected in Table 1. For comparison, the result obtained for gold bound to C27 are shown.

The analysis of the calculated formation energies reveals a very high stabilization of the Au(i) systems with 531 electrons with estimated values of −480 to −530 kJ mol^−1^ depending on the position of the boron. Equally, the binding to carbon in the B-doped graphene fragments seems to be significant, with a predicted binding energy of −420 kJ mol^−1^. The diamagnetic 532-electron molecules exhibit the predicted binding energies in the range of −95 to *ca.* −170 kJ mol^−1^. Taking into account that the diamagnetic 532-electrons system may be considered both as the neutral-ligand Au(0) and negative ligand Au(i) complexes, implies that binding of Au(i) is preferred for the neutral B-doped graphene rather than for the negatively-charged graphene. The triplet 532 electron molecules showed binding energies of roughly 60 kJ mol^−1^ lower than the singlet ones. The values for those are in a similar range as for the 533-electron systems.

**Fig. 5:**
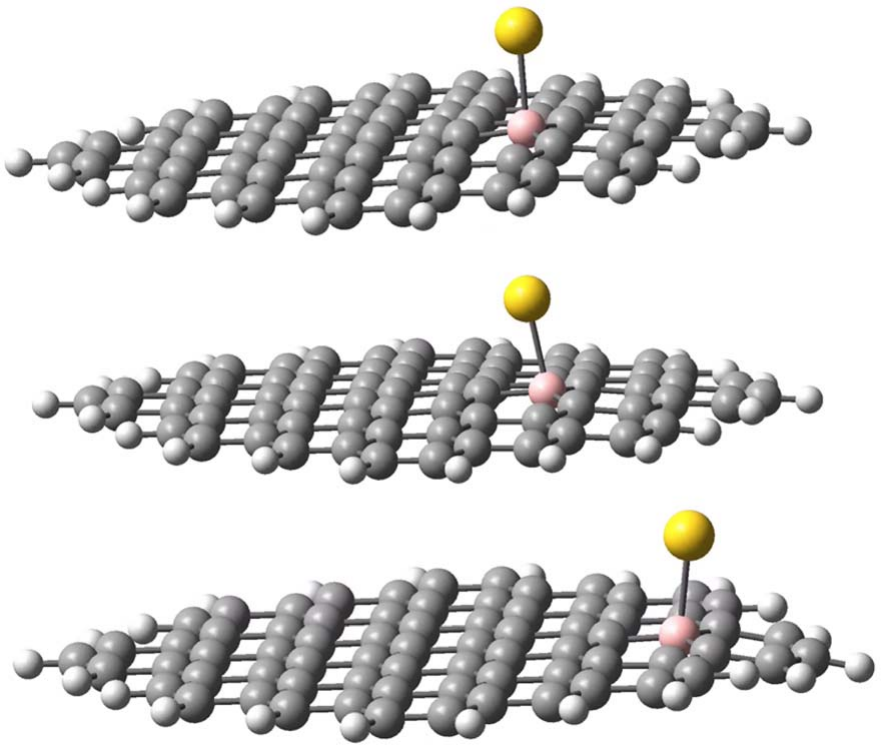
Three types of geometry for Au coordination to B-doped graphene. Top: axial. Middle: B–Au–C. Bottom: bent.

The geometries of the Au B-graphene adducts fall into three categories. The gold atom may be bound axially to boron, forming a bond perpendicular to the graphene plane, with a slightly pyramidal distortion of the BC_3_ fragment. This geometry is the preferred one for the 533-electron molecules. Another geometry corresponds to the Au–B bond forming an angle of more than 100° with the graphene plane and additional bond/contact with the carbon bound to boron. The C–Au length varies from 2.18 Å to 2.56 Å. Finally, for B32 substituted systems, a third mode of coordination was found in which the Au–B bond is again inclined to the graphene plane, but there is no shift of Au towards a particular carbon. Rather, the gold atom is shifted over the six-membered carbon ring. The three modes of coordination are show in Fig. 5.

In the next step, we performed the modelling of gold adducts of the model molecule with an Au_5_ assembly bound to boron in position 30. Similar to the model involving the single Au atom, we considered four possible isomers: (i) 848-electron molecule with a doublet ground state corresponding formally to a negatively-charged C_71_BH_22_ fragment bound to Au_5_(0), (ii) triplet and singlet states of 848-electron molecules corresponding formally to a neutral C_71_BH_22_ fragment bound to Au_5_(0) with the doped-graphene fragment and gold coupled ferromagnetically and antiferromagnetically, respectively. Note that again the diamagnetic isomer may also represent the complex of negatively-charged C_71_BH_22_ bound to (Au_5_)^+1^ cluster, (iii) 531-electron molecule with a doublet ground state, corresponding to the neutral C_71_BH_22_ fragment bound to Au(0). The results are shown in the bottom part of Table 1. Interestingly, while for the 849-electron and triplet 848-electron systems the binding energies lie roughly in the ranges obtained for the single Au-atom models, the respective binding energy for the 847-electron ((Au_5_)^+1^L) system is reduced to *ca.* 50% of that for the single gold complex, while that for diamagnetic 848-electron system is reduced at *ca.* 25% (for complexes involving the boron in position 30). Consequently, for the 848-electron system, the stabilisation of the singlet state with respect to the triplet state is reduced from 55 to 17 kJ mol^−1^.

**Fig. 6:**
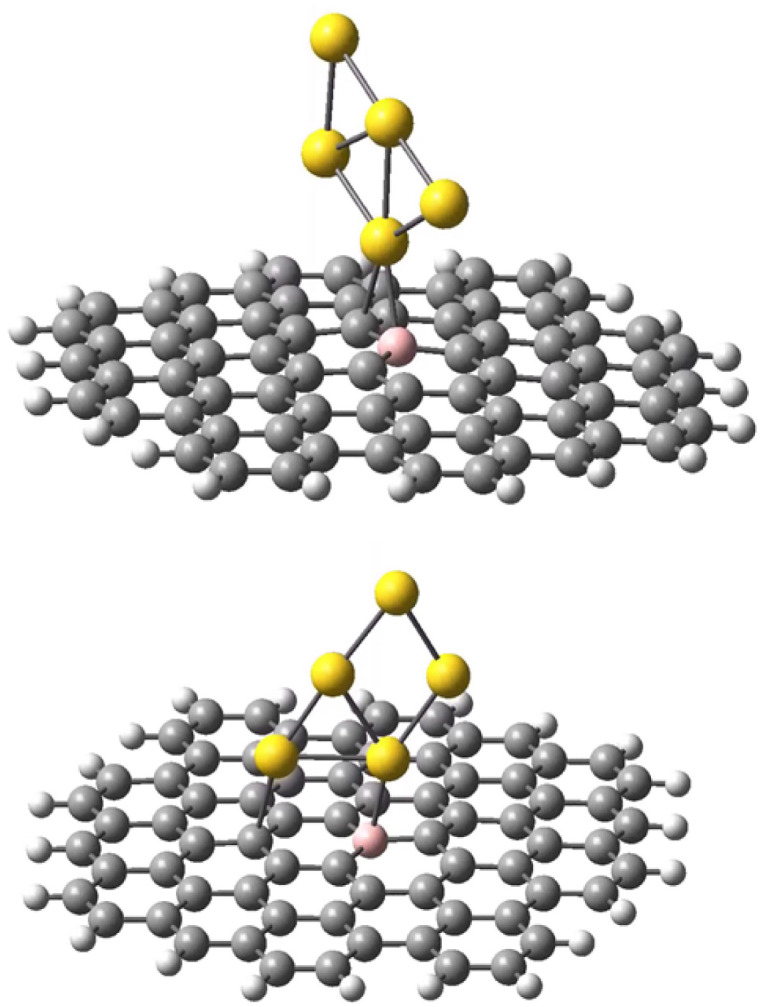
Different modes of Au_5_ binding to B-doped graphene. Top: 848-electron system in the triplet state; bottom: 848-electron system in the singlet state.

In each case, the Au_5_ cluster is bound as a trapezium. Two types of geometries were identified for the Au_5_ complexes. All systems, except for the 848-electron triplet, reveal the trapezium side parallel to the ligand plane with the bonding of one gold atom to boron and another (Au′) to the atom C44, located in *para* position to boron belonging to the same six-membered ring. The Au–B and Au′–C bond distances lie in the range 2.28–2.34 and 2.53–2.59 Å, respectively. For the 848 triplet species, only one gold atom of the trapezium side is coordinated to both B30 and the neighbouring C26 atoms with the respective bond lengths of 2.348 and 2.386 Å. The two geometries are shown in Fig. 6. A similar variety of bonding patterns was found for DFT Au_4_ adducts of graphene models.^33^

#### 2.2.2. B-doped graphene with further defects

**Fig. 7:**
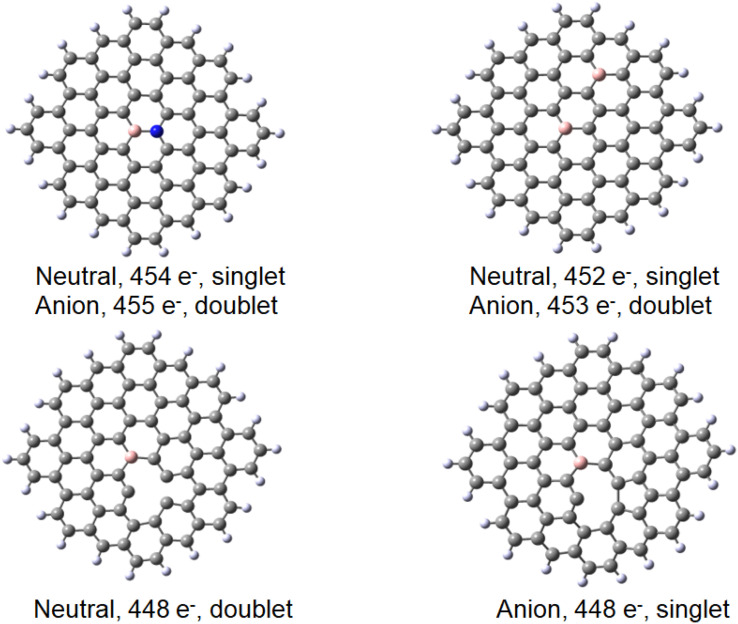
Different modifications of B30C_71_BH_22_ model system used for DFT modelling. The number of electrons and the multiplicity is given for the anion and neutral species. Top: left, B30–N70C_70_BNH_22_, right B30–B47C_70_B_2_H_22_. Bottom, the neutral (left) and anionic (right) forms of the defected C_70_BH_22_. Note that the reference molecule C_71_BH_22_ has 453 electrons in the neutral doublet state and 454 electrons in the anionic singlet state.

Finally, we considered the effects of further modifications of the B-doped graphene. First, taking into account that the systems described in this paper contain nitrogen, we considered the systems with B30 graphene containing the nitrogen atom in position 70 (see Fig. 7, top left). This system corresponds to the reference B30–C_71_BH_22_ with one more electron. On the other hand, the C_70_B_2_H_22_ system in which one carbon of the B30–C_71_BH_22_ is replaced with boron corresponds to one with one electron less. We chose the isomer with the remote B atoms in positions 30 and 47 (Fig. 7, top, right). The system with neighbouring borons in positions 30 and 70 binds the gold with both B atoms (see ESI‡) and is, therefore, less relevant to compare with the system under consideration.

Finally, a defect structure with a vacancy at the position of C27 was considered. Different defects, including vacancies are known to change the electronic and structural properties of graphene.^34^ It seemed therefore of interest to look at the bonding properties of such a structure in comparison to a non-defected one. The structures of the defected B30C_70_BH_22_ are shown in Fig. 7. Both the structure on the anion and of the neutral species are shown, as in this case they differ significantly. For the neutral system, the two carbon atoms at the defect come closer to each other to form a strained five-membered ring with a corresponding C–C distance of 1.82 Å. The graphene ring stays planar. For the anionic species, the formation of the five-membered ring is completed (with a respective C–C distance of 1.62 Å) leading to the folding of the graphene structure (see the pdb defect_ligB30_anion and defect_ligB30_neutral files in ESI‡) corresponding to experimentally observed effects using TEM technique.^35^ The results for the “defected” structures were obtained using the above-mentioned TPSS/QZVP method, this time with Grimme's dispersion correction.^36^ The results for the corresponding Au(0)L^−^, Au(0)L and Au(i)L systems are collected in Table 2 including the data for the reference B30–C_71_BH_22_ system. It should be noted that the application of dispersion correction does not influence the geometries obtained (the Au–B bonds do not differ by more than 0.005 Å), while all the energies obtained for the adducts formed are higher by 30 kJ mol^−1^.

Importantly, when comparing the different systems, the Au(0)L^−^, Au(0)L and Au(i)L molecules may be of different multiplicities that may make the comparison of the energy values more complex. For example, while the B30-model of the Au(0)L^−^ system has one unpaired electron, its analogue with additional nitrogen may be either in singlet or triplet states (Fig. 7).

**Table 2:** Calculated energies and bond distance of Au–B bond formation for the modelled AuC_71_BH_22_ species with a defect in the graphene fragment using the TPSS/qzvp modelling including the dispersion correction. The values for the reference AuC_71_BH_22_ are given

System	Energy Au-adduct formation (kJ mol^−1^)				Geometry of coordination and bond distances (Å)								
	Molecule, number of electrons, spin state				Geom.	Au–B		Geom.	Au–B	Geom.	Au–B	Geom.	Au–B
	Au(0)L^−^ 533 doublet	Au(0)L22 532 triplet	Au(0)L 531 singlet	Au(i)L 530 doublet	Au(0)L^−^ 533 doublet			Au(0)L 532 triplet		Au(0)L 531 singlet		Au(i)L 530 doublet	
B30	−102	−95	−150	−529	Axial		2.292	Axial	2.277	Axial	2.192	Axial	2.188
B_2_30-47	Au(0)L^−^ 532 triplet	Au(0)L^−^ 532 singlet	Au(0)L 531 doublet	Au(i)L 530 singlet	Au(0)L^−^ 532 triplet			Au(0)L^−^ 532 singlet		Au(0)L 531 singlet		Au(i)L 530 singlet	
	−94	−150	−155	−527	Linear		2.290	Linear	2.204		2.194	Linear	2.188
B30–N70	Au(0)L^−^ 534 triplet	Au(0)L^−^ 534 singlet	Au(0)L 533 doublet	Au(i)L 530 singlet	Au(0)L^−^ 534 triplet			Au(0)L^−^ 534 singlet		Au(0)L 533 doublet		Au(i)L 530 singlet	
	−89	−120	−82	−470	Skewed		2.295	Skewed	2.238	Skewed	2.283	Skewed	2.201
Vacancy 27	Au(0)L^−^ 527 doublet	Au(0) 526 triplet	Au(0)L 526 singlet	Au(i)L 525 doublet	Au(0)L^−^ 527 doublet			Au(0) 526 triplet		Au(0)L 526 singlet		Au(i)L 525 doublet	
	−122	−142	−147	−534	Axial^a^		2.151	Axial^a^	2.22.1792	Au–C34– B–C 2.282	Axial^a^	Axial^a^	2.185

a The geometry corresponding to the trigonaly pyramidal Au-BC_3_ fragment in a folded graphene moiety.

**Fig. 8:**
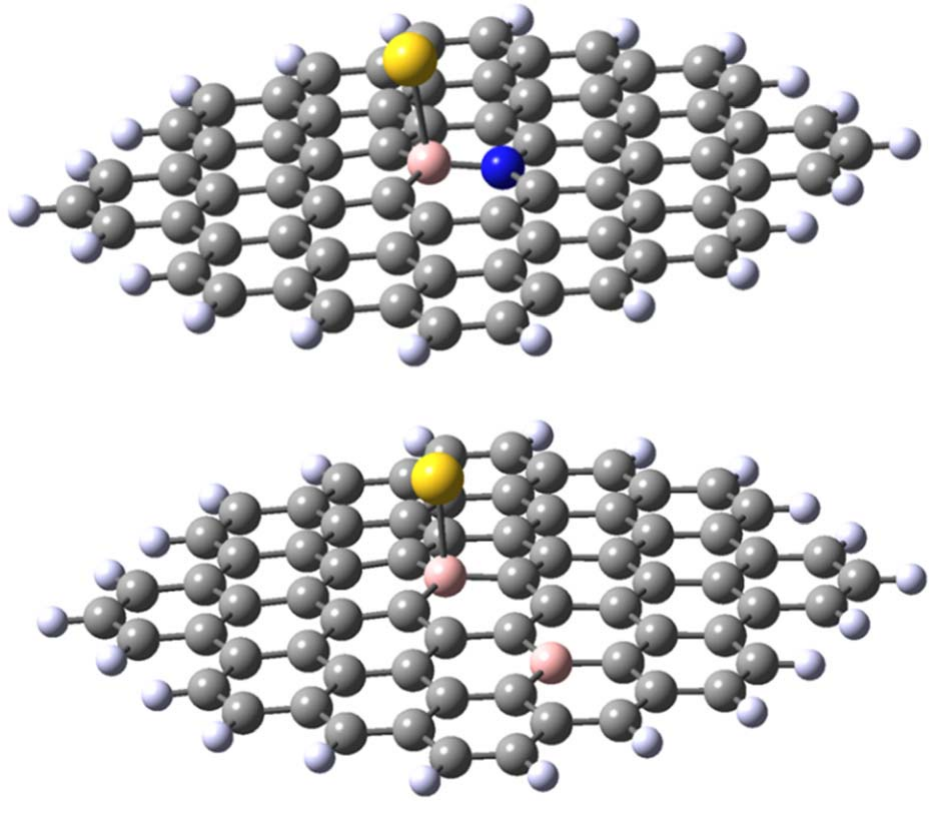
Representative structures of B30–N70 C_70_BNH_22_ (top) and B30–B47 C_70_B2H_22_ (bottom).

The results obtained for the N-doped B-30 substituted molecule allow the following conclusions. Firstly, the energy of adduct formation for Au(0)L and Au(i)L is significantly (60–70 kJ mol^−1^) lower than those for the non-doped B-30 reference molecules with the exception of the triplet Au(0)L spin isomer of B-30 system, that reveals a formation energy higher by only 13 kJ mol^−1^. The corresponding Au–B bond lengths are larger for the N-doped molecules, with the difference for the Au(0)L below 0.1 Å On the other hand for the Au(0)L^−^ adducts the energy of formation of the doublet of the reference molecule lies between those of the singlet and triplet states of its N-doped analogue (−102, compared to −120 and −89 kJ mol^−1^, respectively.). Also, the Au–B bond length for the doublet of the reference molecule lies between these for the singlet and triplet isomers of the N-doped one (2.292 *vs.* 2.295 and 2.238 Å). Noteworthy, however is the clear change of the geometry of the B–Au binding. In all adducts of the N-doped graphene analogue, the DFT optimisation indicates that the B–Au is skewed relative to the graphene plane, yielding N–B–Au angles of 104–106° (see Fig. 8 and pdb files Au_532_ligN_B30, Au_533_ligN_B30, Au_singlet_534_ligN_B30 and Au_triplet_534_ligN_B30 in ESI‡). This may indicate either a repulsive interaction between the Au atom and a lone pair of electrons on nitrogen or/and the electron-rich part of the molecule. The results obtained for the 30,47-B-C_70_B_2_H_22_ system with the second boron atom remote from the B30 centre seem to indicate the importance of the nitrogen low-pair repulsion effects. Although the system contains two electrons less than the reference B30 molecule the formation energies of the Au(0)L and Au(i)L systems are very similar (−529 and −527 kJ mol^−1^ for the reference and B-doped Au(i)L species, and −150 and −155 kJ mol^−1^ (singlet) for the respective Au(0)L analogues). For the Au(0)L^−^ species again the formation energy of the reference molecule (−102 kJ mol^−1^) lies between those of the triplet and singlet states of the B-doped analogue (−94 and −150 kJ/respectively). The bond lengths do not reveal a significant difference compared to those in the isomers of the reference B30–C_71_BH_22_ species, particularly when the differences in the multiplicities of the species with the same formal charge of Au and graphene moiety are taken into account.

**Fig. 9:**
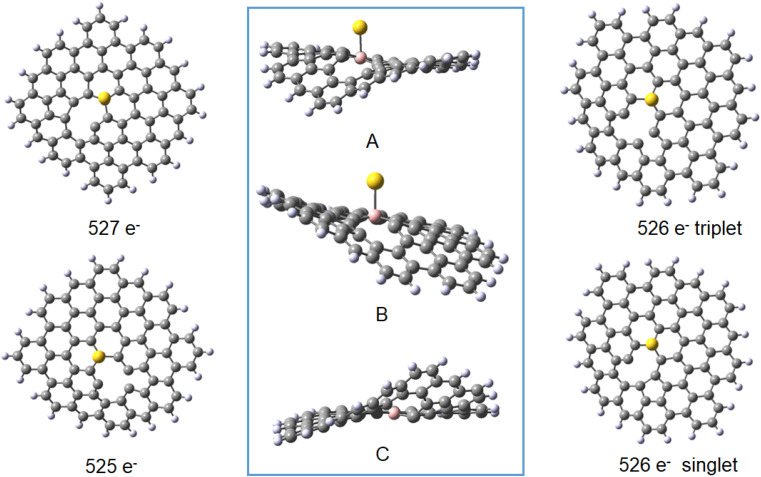
The optimised structures of the 527 electron (Au(0)L^−^, top left), 526 electron (Au(0)L, triplet and singlet, right, top and bottom, respectively) and 525 electron (left, bottom) species of vacancy system B30–C_70_BH_22_. The inset shows the molecules with 525–526 electrons (A) and 527 electrons (B) with the same orientation of the Au–B–C26 atoms. The optimised structure of the anionic ligand is also shown in the inset with the same alignment of B and C26 (C).

The results obtained for the systems with a vacancy in position 27, *i.e.* lacking one carbon in the graphene moiety, may be directly compared to those for the reference B30–C_71_BH_22_ system. While containing six electrons less, the systems reveal the same mulitplicities for all formal charges of gold and graphene. The calculated formation energies point towards some additional stabilisation of the species with a defect in graphene for the Au(0) systems, while the defects seem not bring about significant energetic effects compared to a reference system. The obtained structures shown in Fig. 9 display the difference between the graphene deformations for the 526/527 and 525 species (see the pdb files Au_defect_527, Au_defect_525, Audefect_silnglet_526and AU_defect_527_triplet). Although the isolated neutral and anionic B30–C_70_BH_22_ defected graphene models reveal the different geometries (*vide supra*), they both form differently folded structures when bound to a gold atom. The 526/525 electron species (Au(0)L) and Au(i)L, respectively) reveal the five-membered ring due to formation of the C46–C73 bond corresponding to the structure of the folded anionic free graphene species. On the other hand, the 527 electron form (formally Au(0)L^−^) displays the five-membered ring formed by the bond between C42 and C46 (note that C42 and C73 atoms are not equivalent for the B30 species). This effect implies that the overall charge on Au–B-doped-graphene with a defect influences the localisation of the defect. An interesting feature is that, contrary to all Au–B species discussed before, the Au–B bond length for the triplet Au(0)L species is shorter than that for the singlet state. Additionally, the latter reveals a different geometry with an Au–B34 distance of 2.304 Å and C–B–Au angle of 72°. This together with the comaprable formation energies of the singlet and triplet states (−147 and −142 kJ mol^−1^, respectively) may suggest that the singlet-state form may correspond to the previously mentioned possibility that the complex is a negatively-charged BC_70_BH_22_ bound to Au(i), isolectronic with that of Au(0) bound to the neutral ligand. Comparison of bond lengths with the reference B30–C_71_BH_22_ indicates a pronounced difference of 0.15 Å between them, with that of the “defected” Au(0)L species revealing only a 2.151 Å Au–B bond.

In summary, DFT modelling of the interactions of gold with the doped graphene suggests the possibility of formation of Au–B bonds with strengths higher than those for Au–C in the same systems, reveals the effects of doping the graphene with N- and B-defects for the geometry of Au-bonding, and shows pronounced geometrical effects brought about in the systems with a carbon vacancy. The change of the affinity for gold binding by introducing a vacancy is dependent on the overall charge of the system and is pronounced for the anionic species.

## 3. Conclusions

Recent advances in aberration-corrected transmission electron microscopy (TEM) and scanning transmission electron microscopy (STEM) provide an opportunity for imaging ultra-dynamic systems in real-time. However, as yet, there are few reports of studies of such dynamic processes at the single-atom level. Here, we synthesized two 16e square-planar bis-thiolato-Au(iii) complexes, [Au^III^(1,2-dicarba-*closo*-dodecarborane-1,2-dithiolato)_2_][NBu_4_] (Au-1) and [Au^III^(4-methyl-1,2-benzenedithiolato)_2_][NBu_4_] (Au-2). Au-1 and Au-2 differ only in the presence of carboranyl or tolyl groups as substituents on chelated 1,2-dithiolato ligands. Au-1/Au-2 was then encapsulated in the symmetrical triblock copolymer poloxamer (Pluronic®) P123 containing blocks of poly(ethylene oxide) and poly(propylene oxide) to prepare the micelles AuMs-1 and AuMs-2. Then, we generated single gold atoms and gold nanocrystals on S-doped boronic graphitic surfaces or S-doped amorphous carbon surfaces by electron irradiation (using scanning transmission electron microscopy (STEM)) of AuMs-1 and AuMs-2. The boron doping appears to create anchoring sites for Au atoms. EELS data, along with DFT calculations, suggested the presence of unprecedented gold–boron interactions within the nanomaterial. Although Au–B bonds within pure Au–B alloys^19^ or in gold boride^20^ have been reported, direct Au–B interactions appear to be unknown in nanomaterials.

This strategy has potential for controlling the dynamics of metal atoms on fabricated matrices by suitable choice of metals and ligands, and might lead to next-generation nano-devices with a wide range of applications. There is also potential for extending such fabrication to novel nano-alloys. The DFT modelling reveals that the defects, particularly vacancies, may affect the binding of gold atoms to non-ideal graphenic materials containing boron.

## Conflicts of interest

There are no conflicts to declare.

## Supplementary Material

NA-006-D3NA00956D-s001

NA-006-D3NA00956D-s002
