# Supplementary material for: Experimental and theoretical evidence for unprecedented strong interactions of gold atoms with boron on boron/sulfur-doped carbon surfaces
Source: Nanoscale Adv. 2023 Dec 11;6(7):1837–46. doi: 10.1039/d3na00956d (PMC10964758; doi:10.1039/d3na00956d)
Supplement: NA-006-D3NA00956D-s001 [file NA-006-D3NA00956D-s001.pdf]

Electronic Supplementary Material (ESI) for Nanoscale Advances.
This journal is © The Royal Society of Chemistry 2023

# Electronic Supplementary Information (ESI†)

## Experimental and theoretical evidence for unprecedented interactions of boron with gold atoms on boron/sulfur-doped carbon surfaces

Samya Banerjee,*[a][b] Juliusz A. Wolny,*[c] Mohsen Danaie,*[d] Nicolas P. E. Barry,#[a][e] Yisong Han,[f] Houari Amari,[f] Richard Beanland,[f] Volker Schünemann,[c] and Peter J. Sadler*[a]

[a] Dr. Samya Banerjee and Prof. Peter J. Sadler
Department of Chemistry, University of Warwick, CV4 7AL Coventry, U.K
E-mail: P.J.Sadler@warwick.ac.uk

[b] Dr. Samya Banerjee
Department of Chemistry, Indian Institute of Technology (BHU), Varanasi, Uttar Pradesh, 221005, India. Email: samya.chy@itbhu.ac.in

[c] Dr. Juliusz A. Wolny and Prof. Volker Schünemann
Department of Physics, University of Kaiserslautern-Landau, Erwin-Schrödinger-Straße 46, 67663 Kaiserslautern, Germany
E-mail: wolny@rhrk.uni-kl.de

[d] Dr. Mohsen Danaie
Diamond Light Source Ltd, Harwell Science & Innovation Campus, Didcot, Oxfordshire OX11 0DE, U.K
E-mail: mohsen.danaie@diamond.ac.uk

[e] Prof. Nicolas P. E. Barry
School of Chemistry and Biosciences, University of Bradford, Bradford BD1 7DP, U. K.

[f] Dr. Yisong Han, Dr. Houari Amari and Prof. Richard Beanland
Department of Physics, University of Warwick, Gibbet Hill Road, Coventry CV4 7Al, U. K.

# Passed away during the preparation of the manuscript.

## Contents


1. **Materials and Methods**
2. **DFT Modelling of the systems with B-B bond**
3. **Figures S1-S9**

**Figure S1.** Representative STEM image showing single Au atoms and Au-crystals generated from **AuMs-1** upon electron beam irradiation.

**Figure S2.** (a) Gold crystal along with inter-Au atoms distances (in nm) (right) generated from **AuMs-1** upon 5 min electron beam irradiation. (b) FFT of the Au nanocrystal shown in (a, yellow box).

**Figure S3.** EELS spectra showing the presence of sulfur, boron and carbon in a graphitic surface generated from **AuMs-1**.

**Figure S4.** EELS spectra showing involvement of $sp^2$ hybridized carbon in the graphite like matrices formed from **AuMs-1**.

**Figure S5.** Principal component analysis (using nonnegative matrix factorization algorithm; NMF) of EELS data showing the simultaneous presence of C, B in the graphitic matrices.

**Figure S6.** DLS data for **AuMs-2** (1 mg $mL^{-1}$, $H_2O$).

**Figure S7.** Image of Au nanocrystals generated from **AuMs-2** upon electron beam irradiation with sizes ranging from 0 (single atoms) to ~ 2.7 nm, along with Au-Au inter-atomic distances (right, in nm) for the boxed crystal.

**Figure S8.** Size distribution for Au nano-crystals generated from **AuMs-2** upon electron beam irradiation (80 keV) for 5 min.

**Figure S9.** Distribution of Au-Au distances for Au-nanocrystals generated from **AuMs-2**.

**Figure S10.** Model graphene system with B-B bond.

**References**

**List of PDB files available as ESI**

## 1. Materials and Methods

### Materials:

$NaAuCl_4$•$2H_2O$, toluene-3,4-dithiol, Pluronic® P123, anhydrous tetrahydrofuran, methanol were all purchased from Sigma-Aldrich and used as received. Triethylamine was purchased from Fisher Scientific, and dialysis membranes from Spectrum Laboratories, Inc., Spectra/Por®6; MWCO 1 kDa (flat width 45 mm, diameter 29 mm, vol/length 6.4 mL/cm). Pure water (18.2 MΩ) was from a Purelab UHQ USF Elga system. Lacey carbon copper grids with 200 mesh were purchased from Agar Scientific. The synthesis of the complexes [$Au^{III}$(1,2-dicarba-*closo*-dodecarborane-1,2-dithiolato)$_2$][$NBu_4$] (**Au-1)** and [$Au^{III}$(4-methyl-1,2 benzenedithiolato)$_2$][$NBu_4$] (**Au-2)** were based on earlier reports.[1,2]

### Synthesis of AuMs-1 and AuMs-2:

A dry tetrahydrofuran (THF) solution (1 mL) of complex **Au-1** or **Au-2** (5 mg/mL) was added to 10 mL of an aqueous solution of the polymer P123 (5 mg/mL), and the resultant mixture was stirred at ambient temperature for 5 h. The solution was dialyzed to remove the THF (MWCO = 1000Da), for 48 h, and then freeze-dried to give **AuMs-1** or **AuMs-2**.

### Dynamic light scattering (DLS):

The hydrodynamic diameter ($D_h$) of **AuMs-2** was determined by DLS. An aqueous solution of **AuMs-2** (1 mg $ml^{-1}$) was studied on a Malvern Zetasizer NanoS instrument with a 4 mW He–Ne 633 nm laser module at 298 K. Measurements were made at 173° detector angle. Data were analyzed by Malvern DTS 6.20 software. $D_h$ was calculated by fitting the apparent diffusion coefficient in the equation $D_h = kT/(3\pi\eta D_{app})$, where k = the Boltzmann constant, T = temperature, and η = viscosity of the water.

### Transmission Electron Microscopy

TEM experiments were performed on a JEOL JEM-2100 plus microscope operated at 200 keV.

### Scanning Transmission Electron Microscopy

High-resolution scanning electron microscopy was performed on a probe aberration-corrected JEOL ARM 200F, operated at 80 keV accelerating voltage. For imaging, we used 40 µm probe-forming aperture resulting in 30.5 mrad probe convergence half-angle and high-angle annular

dark-field detector at 4 cm camera length, collecting scattered electrons between 120 and 410 mrad. This microscope is equipped with a Quantum GIF for electron energy loss spectroscopy (EELS). EELS spectrum images were acquired at similar optical conditions, with a probe current of 88 pA (spot size 5C). Initially the scanning electron beam caused significant buildup of contamination on the sample. This was mitigated by illuminating a large area of the sample with the 150 µm probe-forming aperture and highly defocused sample with a stationary beam of around 9.6 nA for 5 min. This illumination period was also used to trigger electron beam-induced structural changes in the micelles.

## 2. DFT Modelling of the systems with B-B bond

Here, the results for another "defected" graphene system containing boron are given. We considered the system boron atoms in positions 30 and 70 of the used graphene model that is shown in Figure S10. Following the reasoning in the main text the anionic and neutral forms may bind to Au(0) and Au(I) forming the systems listed in Table below.

| Molecule, number of electrons, spin state | | | | | | | | |
|---|---|---|---|---|---|---|---|---|
| $B_2$30-70 | $Au(0)L^-$<br>532<br>triplet | | $Au(0)L^-$<br>532<br>singlet | | Au(0)L<br>531<br>singlet | | Au(I)L<br>530<br>singlet | |
| Geometry of the Au-bonding and B-Au distances (Å) | | | | | | | | |
| | linear | 2.290 | linear | 2.204 | | 2.194 | linear | 2.188 |
| | chelate $C_s$ | 2.240 | chelate $C_s$ | 2.237 | chelate $C_s$ | 2.230 | chelate $C_s$ | 2.223 |

In all cases the gold is bound to two borons forming the equidistant bonds. The representative geometry is in Figure S12 for the 530 electron systems.

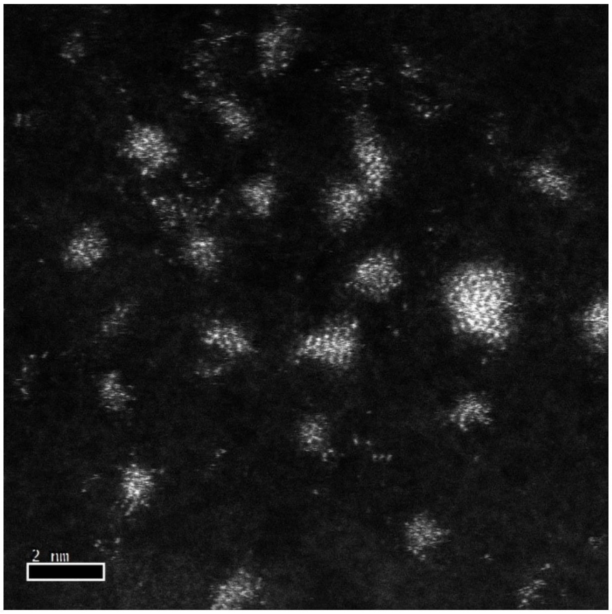


**Figure S1.** Representative STEM image showing single Au atoms and Au-crystals of different sizes generated from **AuMs-1** upon electron beam irradiation.

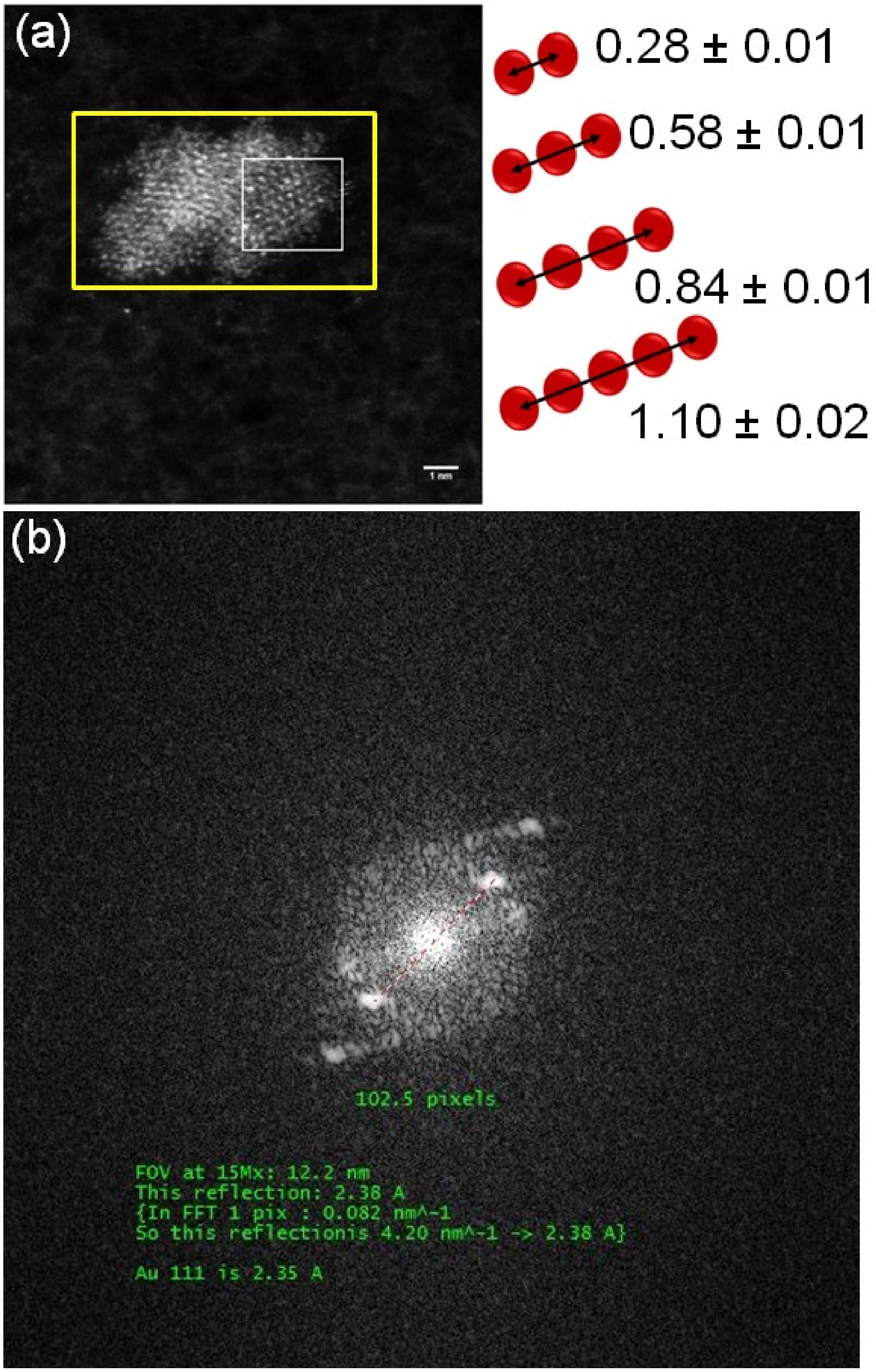


**Figure S2.** (a) Gold crystal along with inter-Au atoms distances (in nm) (right) generated from **AuMs-1** upon 5 min electron beam irradiation. (b) FFT of the Au nanocrystal shown in (a, yellow box).

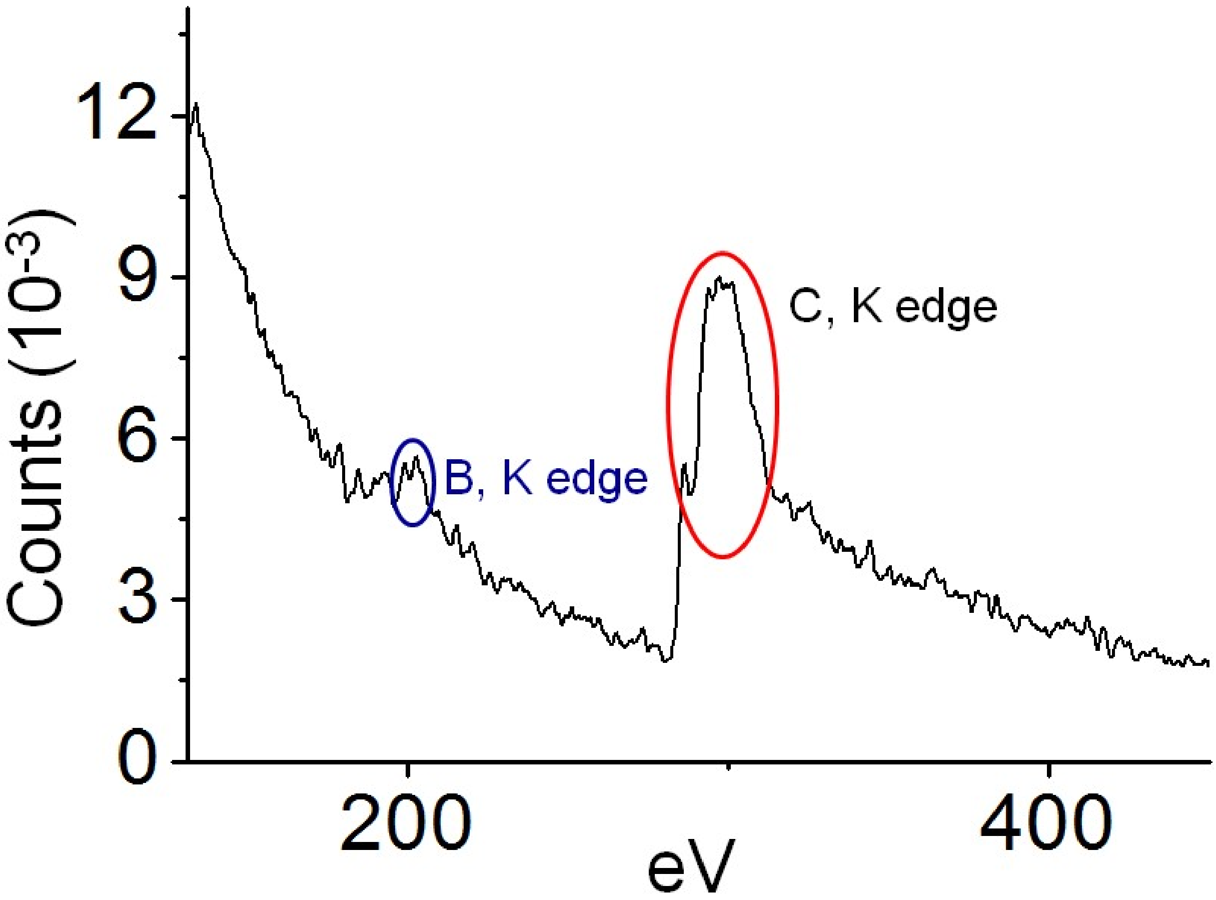


**Figure S3.** EELS spectra showing the presence of sulfur, boron and carbon in a graphitic surface generated from **AuMs-1**.

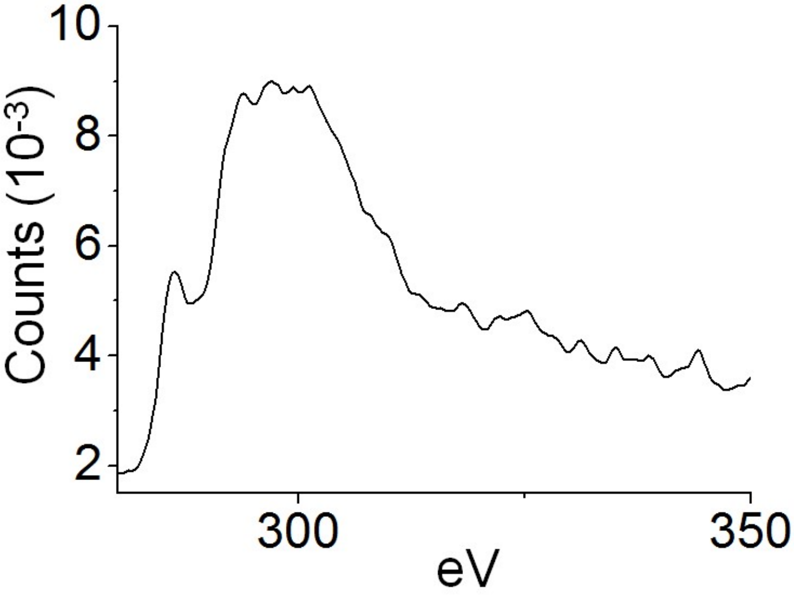


**Figure S4.** EELS spectra showing involvement of $sp^2$ hybridized carbon in the graphite like matrices formed from **AuMs-1**, evident from the C K-edge with a sharp π* feature at ca 286 eV.

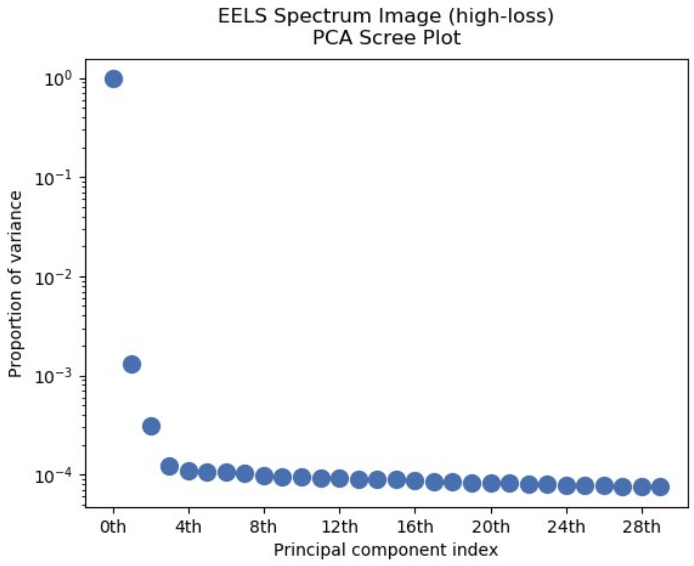
EELS Spectrum Image (high-loss)
PCA Scree Plot
Proportion of variance
$10^0$
$10^{-1}$
$10^{-2}$
$10^{-3}$
$10^{-4}$
0th
4th
8th
12th
16th
20th
24th
28th
Principal component index

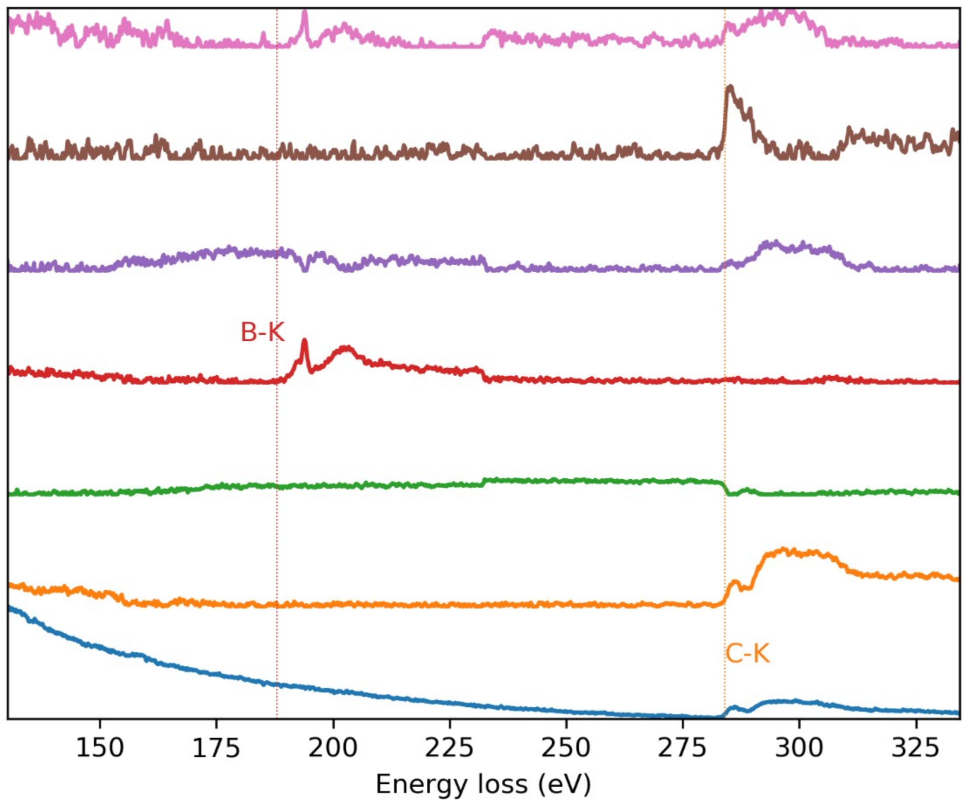
B-K
C-K
150
175
200
225
250
275
300
325
Energy loss (eV)

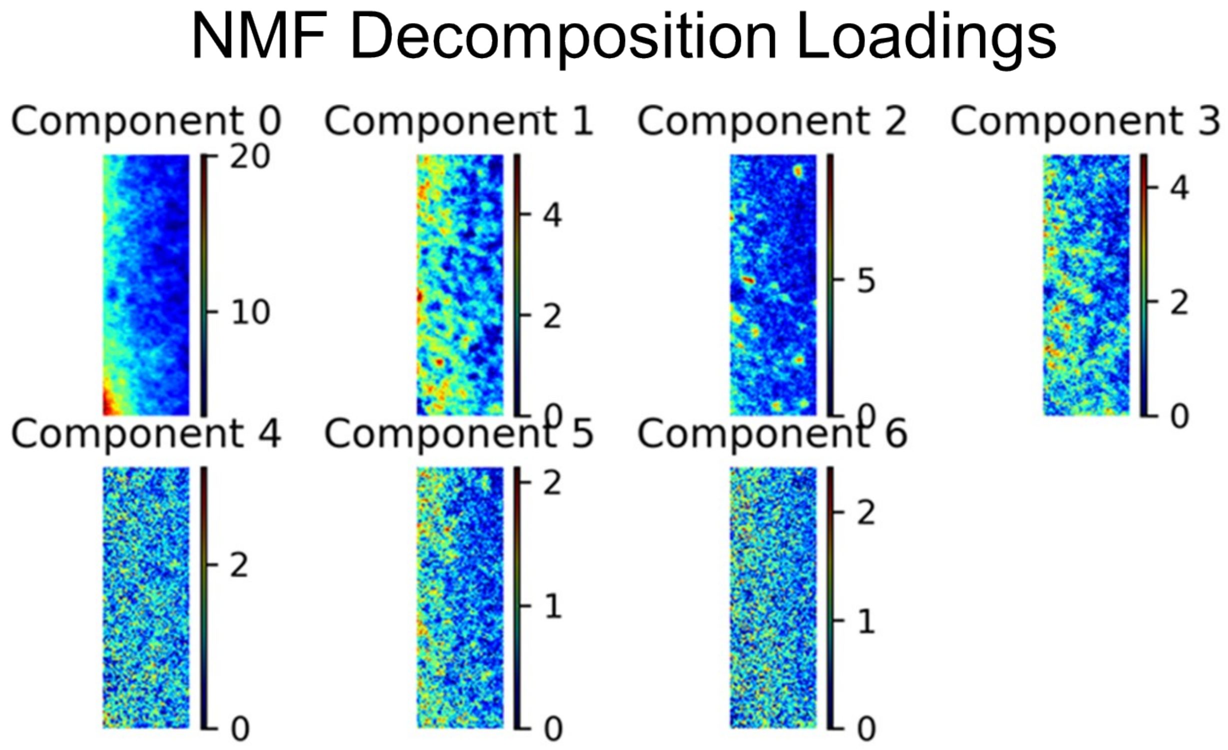


**Figure S5.** Principal component analysis (using nonnegative matrix factorization algorithm; NMF) of EELS data showing the simultaneous presence of C, B in the graphitic matrices. The "scree plot" (top) obtained from the PCA provides an indication of how many components contribute the most to the EELS data. The first component is the average of the dataset. Component-2 is for the C-K edge. The B-K edge is captured in component-4, Components 5 and 6 both have C-K edge features and Component 7 is mostly noise. Notably the density of B is higher in the regions rich in Au nanocrystals.

.

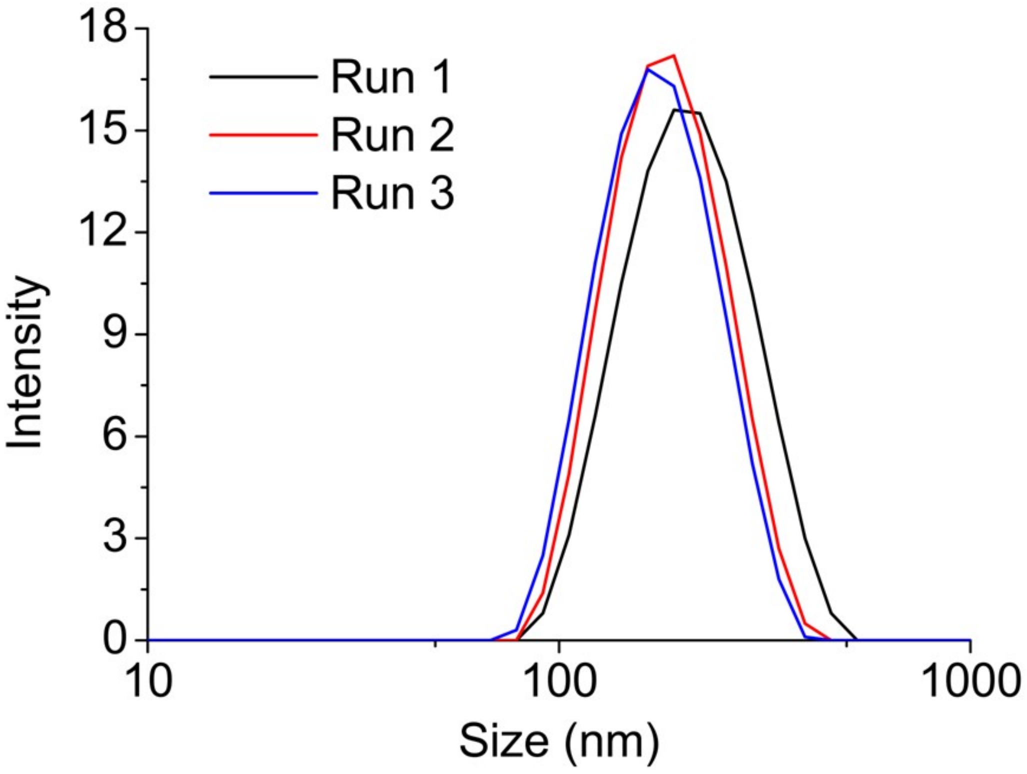


**Figure S6.** DLS data for **AuMs-2** (1 mg mL$^{-1}$, $H_2O$).

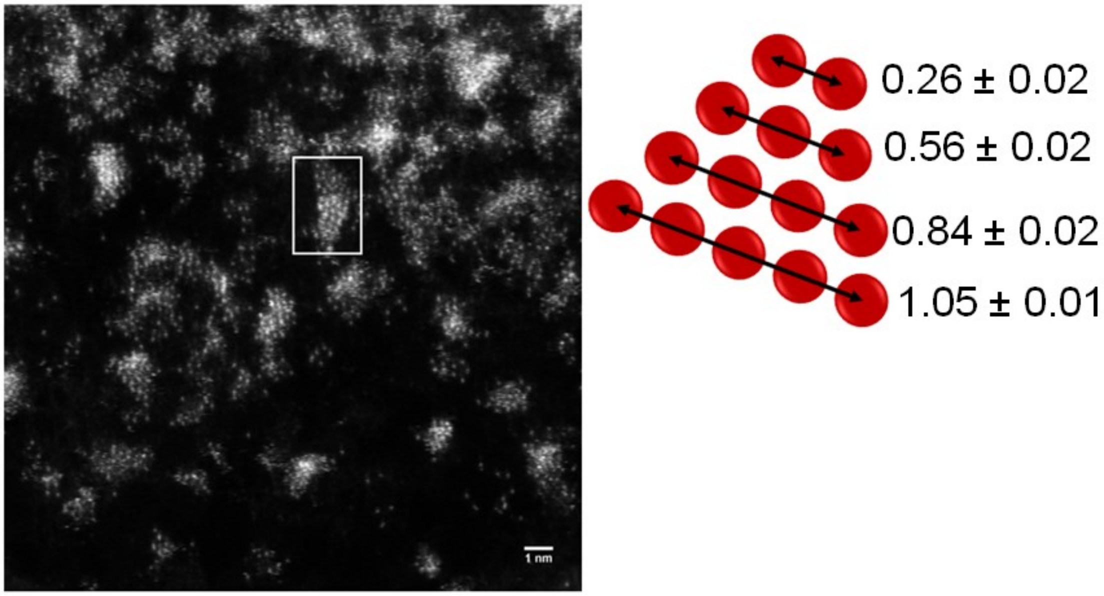


**Figure S7.** Image of Au nanocrystals generated from **AuMs-2** upon electron beam irradiation with sizes ranging from 0 (single atoms) to ~ 2.7 nm, along with Au-Au inter-atomic distances (right, in nm) for the boxed crystal.

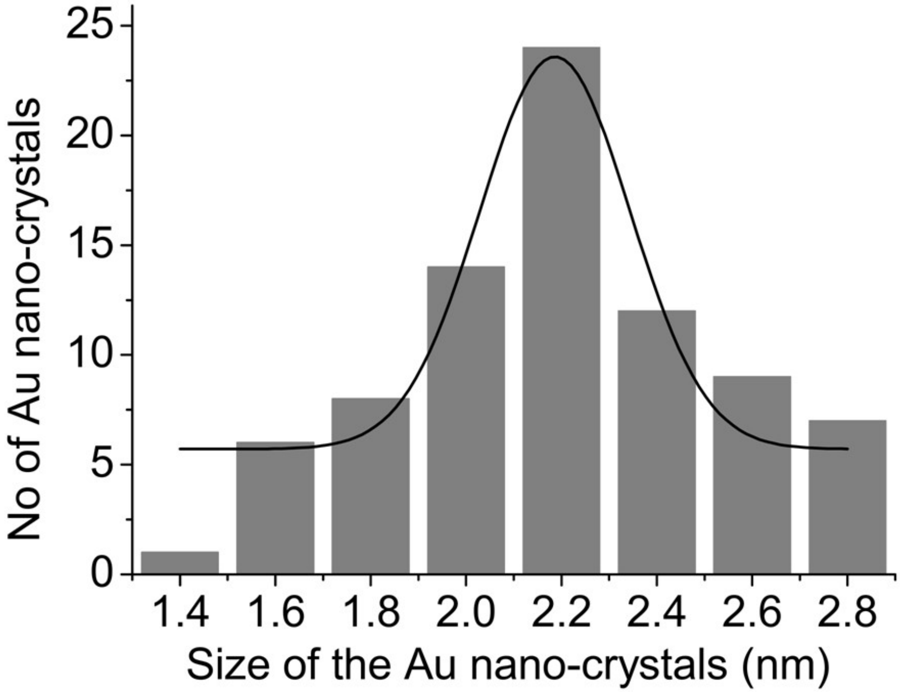


**Figure S8.** Size distribution for Au nano-crystals generated from **AuMs-2** upon electron beam irradiation (80 keV) for 5 min.

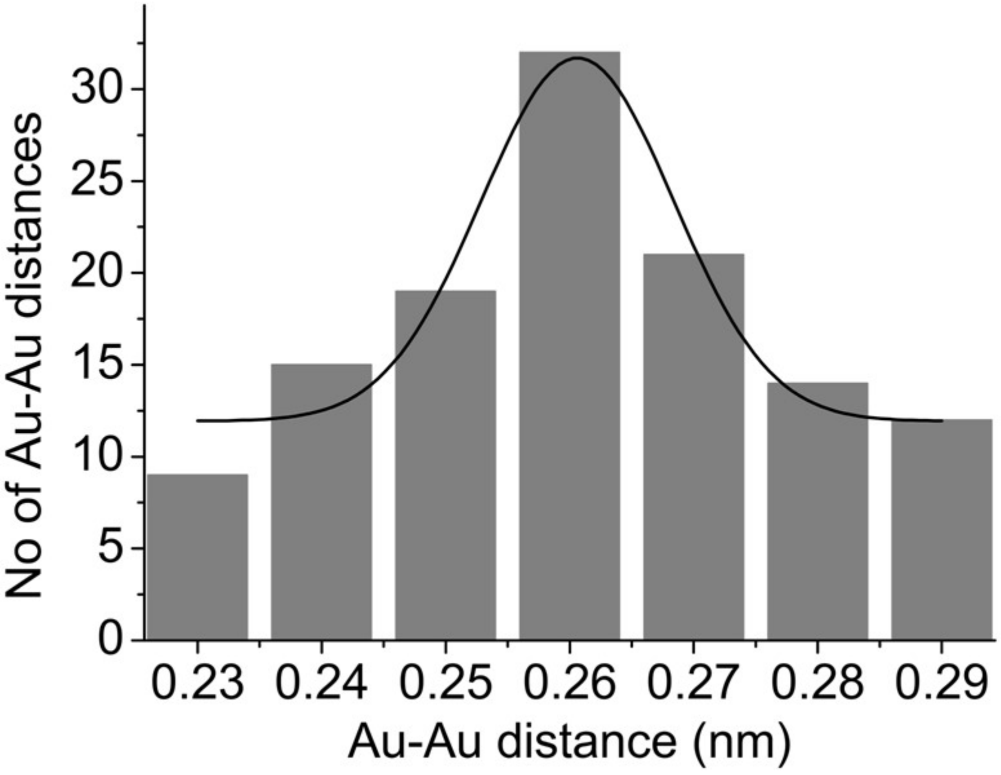


**Figure S9.** Distribution of Au-Au distances for Au-nanocrystals generated from **AuMs-2**.

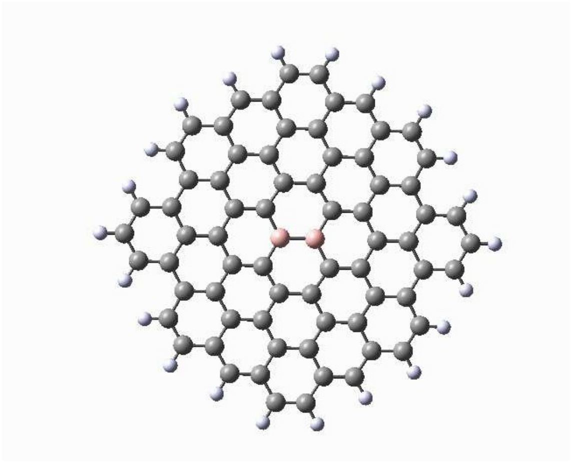

**Figure S10.** Model graphene system with B-B bond.

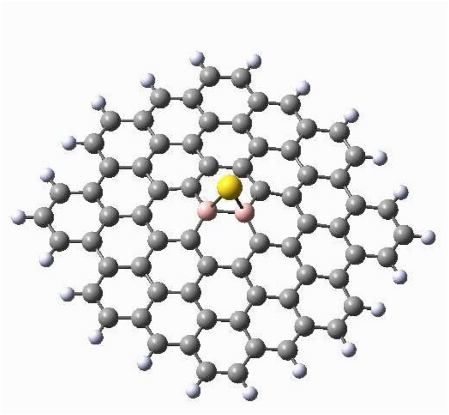

**Figure S11.** The representative structure of the Au-$B_2C_{70}H_{22}$ molecule.

**References:**


3. O. Crespo, M. C. Gimeno, P. G. Jones and A. Laguna, *J. Chem. Soc.*, *Dalton Trans.*, 1997, 1099–1102.
4. M. A. Mazid, M. T. Razi and P. J. Sadler, *Inorg. Chem.* 1981, **20**, 2872-2877.


**List of PDB files available as ESI**

| **File name** | **Description** |
|---|---|
| B30_531_doublet | B30-AuC71BH22, Au(I)L |
| B30_532_diamagnetic | B30-AuC71BH22, Au(0)L |
| B30_533_doublet | B30-AuC71BH22, Au(0)$L^-$ |
| B30_Au5_848_diamagnetic | B30-$Au_5$C71BH22, Au(0)L singlet |
| B30_Au5_848_triplet | B30-$Au_5$C71BH22, Au(0)L triplet |
| | |
| Au_532_ligN_B30 | B30-N70 C70BNH22 Au(I)L |
| Au_533_ligN_B30 | B30-N70 C70BNH22 Au(0)L |
| Au_singlet_534_ligN_B30 | B30-N70 C70BNH22 Au(0)$L^-$ singlet |
| Au_triplet_534_ligN_B30 | B30-N70 C70BNH22 Au(0)$L^-$ triplet |
| | |
| Au_defect_527 | B30-C70BH22 Au(0)$L^-$ |
| Au_defect_singlet_526 | B30-C70BH22 Au(0)L singlet |
| Au_defect_triplet_526 | B30-C70BH22 Au(0)L triplet |
| Au_def_525 | B30-C70BH22 Au(I)L |
| | |
| defect_ligB30_neutral | Neutral B30-C70BH22 with a vacancy |
| defect_ligB30_anion | Anionic B30-C70BH22 with a vacancy |
