# Supplementary material for: Experimental and theoretical evidence for unprecedented strong interactions of gold atoms with boron on boron/sulfur-doped carbon surfaces
Source: Nanoscale Adv. 2023 Dec 11;6(7):1837–46. doi: 10.1039/d3na00956d (PMC10964758; doi:10.1039/d3na00956d)
Supplement: NA-006-D3NA00956D-s002 [file NA-006-D3NA00956D-s002.zip › suppl_files/sadler-eels-svd.html]

Sadler-EELS-SVD


# Sadler EM19373-2¶

Loading raw data   
Removing spikes and aligning to ZLP   
Principle Component Analysis

In [1]:

```
%matplotlib notebook 
import matplotlib.pyplot as plt
import hyperspy.api as hs
hs.set_log_level('ERROR')
import numpy as np
import os

from IPython.display import HTML, display

import warnings
warnings.simplefilter(action='ignore', category=FutureWarning)
plt.rcParams.update({'figure.max_open_warning': 0})
warnings.filterwarnings("ignore")
#def hide_code():
display(HTML('''<script>
code_show=true; 
function code_toggle() {
 if (code_show){
 $('div.input').hide();
 } else {
 $('div.input').show();
 }
 code_show = !code_show
} 
$( document ).ready(code_toggle);
</script>
<form action="javascript:code_toggle()"><input type="submit" value="Click here to toggle on/off the raw code."></form>'''))

#hide_code()
```

# Loading raw data¶

In [2]:

```
os.chdir(r'/dls/science/groups/e02/Mohsen/Sadler_data/em19373-2/GATAN/A6/Y/SI data (18)/')
```

In [6]:

```
# #load from saved hspy versions
# eels_core = hs.load('eels_core.hspy')
# eels_low = hs.load('eels_low.hspy')
```

In [3]:

```
#######To load the raw data
eels_core = hs.load('EELS Spectrum Image (high-loss).dm4')
eels_low = hs.load('EELS Spectrum Image (low-loss).dm4')
```

In [4]:

```
eels_core.sum().plot()
eels_low.sum().plot()
```

In [5]:

```
eels_core.axes_manager
```

Out[5]:

**< Axes manager, axes: (50, 150|2048) >**

| Navigation axis name | size | index | offset | scale | units |
| --- | --- | --- | --- | --- | --- |
| x | 50 | 0 | -0.0 | 0.20383207499980927 | nm |
| y | 150 | 0 | -0.0 | 0.20383207499980927 | nm |

| Signal axis name | size | offset | scale | units |
| --- | --- | --- | --- | --- |
| Energy loss | 2048 | 130.00000193715096 | 0.10000000149011612 | eV |

# Removing spikes and aligning to ZLP¶

In [9]:

```
#Spike removal 
eels_low.isig[2.7:].spikes_removal_tool()
```

In [11]:

```
eels_core.spikes_removal_tool()
```

In [12]:

```
#Aligning the data wrt the zero-loss peak
eels_low.align_zero_loss_peak(calibrate = True,print_stats = True, also_align=[eels_core])
```

```
Initial ZLP position statistics
-------------------------------
Summary statistics
------------------
mean:	-0.0149
std:	0.0381

min:	-0.1
Q1:	0
median:	0
Q3:	0
max:	0.1
```

In [13]:

```
#After aligning  / spike removal
eels_core.save('eels_core')
eels_low.save('eels_low')
```

In [ ]:

```
###### Skip to here if loading pre-processed data
```

In [14]:

```
eels_core.axes_manager
```

Out[14]:

**< Axes manager, axes: (50, 150|2043) >**

| Navigation axis name | size | index | offset | scale | units |
| --- | --- | --- | --- | --- | --- |
| x | 50 | 0 | -0.0 | 0.20383207499980927 | nm |
| y | 150 | 0 | -0.0 | 0.20383207499980927 | nm |

| Signal axis name | size | offset | scale | units |
| --- | --- | --- | --- | --- |
| Energy loss | 2043 | 130.2000019401312 | 0.10000000149011612 | eV |

In [17]:

```
#Interactive sum spectrum over image roi
eels_core.plot()
rec_roi = hs.roi.RectangularROI(left = 1., right = 1.5, top = 1.9, bottom= 2.0)
roi_reg = rec_roi.interactive(eels_core)
roi_reg_sum = hs.interactive(roi_reg.sum, event= roi_reg.axes_manager.events.any_axis_changed)
roi_reg_sum.plot()
```

# Principal component analysis¶

In [23]:

```
eels_core.plot()
```

In [24]:

```
eels_core.decomposition()
```

```
Decomposition info:
  normalize_poissonian_noise=False
  algorithm=svd
  output_dimension=None
  centre=None
```

In [25]:

```
eels_core.learning_results.summary()
```

Out[25]:

```
'Decomposition parameters\n------------------------\nnormalize_poissonian_noise=False\nalgorithm=svd\noutput_dimension=None\ncentre=None'
```

In [26]:

```
eels_core.plot_explained_variance_ratio()
```

Out[26]:

```
<matplotlib.axes._subplots.AxesSubplot at 0x7f7f123f1f50>
```

In [28]:

```
eels_core.decomposition(algorithm= 'RPCA_GoDec', output_dimension= 4)
```

```
Decomposition info:
  normalize_poissonian_noise=False
  algorithm=rpca
  output_dimension=4
  centre=None
```

In [29]:

```
eels_core.plot_decomposition_results()
```

In [30]:

```
#To avoid nmf error due to negative values
eels_core_pos = hs.signals.EELSSpectrum(eels_core.data.clip(min=0))
```

In [39]:

```
eels_core_pos.decomposition(True, algorithm= 'nmf', output_dimension= 7)
```

```
Decomposition info:
  normalize_poissonian_noise=True
  algorithm=nmf
  output_dimension=7
  centre=None
scikit-learn estimator:
NMF(alpha=0.0, beta_loss='frobenius', init=None, l1_ratio=0.0, max_iter=200,
    n_components=7, random_state=None, shuffle=False, solver='cd', tol=0.0001,
    verbose=0)
```

In [43]:

```
eels_core_pos.axes_manager = eels_core.axes_manager
```

In [44]:

```
eels_core_pos.axes_manager
```

Out[44]:

**< Axes manager, axes: (50, 150|2043) >**

| Navigation axis name | size | index | offset | scale | units |
| --- | --- | --- | --- | --- | --- |
| x | 50 | 0 | -0.0 | 0.20383207499980927 | nm |
| y | 150 | 0 | -0.0 | 0.20383207499980927 | nm |

| Signal axis name | size | offset | scale | units |
| --- | --- | --- | --- | --- |
| Energy loss | 2043 | 130.2000019401312 | 0.10000000149011612 | eV |

In [42]:

```
eels_core.axes_manager
```

Out[42]:

**< Axes manager, axes: (50, 150|2043) >**

| Navigation axis name | size | index | offset | scale | units |
| --- | --- | --- | --- | --- | --- |
| x | 50 | 0 | -0.0 | 0.20383207499980927 | nm |
| y | 150 | 0 | -0.0 | 0.20383207499980927 | nm |

| Signal axis name | size | offset | scale | units |
| --- | --- | --- | --- | --- |
| Energy loss | 2043 | 130.2000019401312 | 0.10000000149011612 | eV |

In [45]:

```
eels_core_pos.plot_decomposition_results()
#Issue with the edge of a detector quadrant
```

In [46]:

```
results_l = eels_core_pos.get_decomposition_loadings()
results_f = eels_core_pos.get_decomposition_factors()
```

In [47]:

```
from matplotlib import pyplot as plt
```

In [84]:

```
hs.plot.plot_images(results_l, cmap = 'jet', per_row=4, suptitle='NMF Decompostion Loadings', axes_decor='off', 
                   label=['Component {}'.format(i) for i in range(results_l.data.shape[0])])
plt.savefig('/dls/science/groups/e02/Mohsen/Sadler_data/em19373-2/GATAN/A6/Y/SI data (18)/eels_nmf_loadings.png', dpi = 300)
```

In [87]:

```
hs.plot.plot_spectra(results_f, style='cascade')

plt.axvline(284, c='C1', ls=':', lw=0.5)
plt.text(x=284, y=0.50, s='C-K$_\\alpha$', color='C1')
plt.axvline(188, c='C3', ls=':', lw=0.5)
plt.text(x=180, y=3.37, s='B-K$_\\alpha$', color='C3')
plt.savefig('/dls/science/groups/e02/Mohsen/Sadler_data/em19373-2/GATAN/A6/Y/SI data (18)/eels_nmf_factors.png', dpi = 300)
```

In [38]:

```
eels_core_pos.save('eels_core_pos')
```

In [54]:

```
eels_core_pos.sum().plot()
```

In [ ]:

```

```
